# Impaired immune responses and prolonged viral replication in lung allograft recipients infected with SARS-CoV-2 in the early phase after transplantation

**DOI:** 10.1007/s15010-023-02116-6

**Published:** 2023-11-03

**Authors:** Olaf M. Glueck, Xiaoling Liang, Irina Badell, Paul R. Wratil, Alexander Graf, Stefan Krebs, Helmut Blum, Johannes C. Hellmuth, Clemens Scherer, Alexandra Hollaus, Patricia M. Spaeth, Burak Karakoc, Thimo Fuchs, Julia Zimmermann, Teresa Kauke, Andreas Moosmann, Oliver T. Keppler, Christian Schneider, Maximilian Muenchhoff

**Affiliations:** 1 Division of Thoracic Surgery, LMU University Hospital, LMU Munich, Munich, Germany; 2 Max Von Pettenkofer Institute and Gene Center, Virology, National Reference Center for Retroviruses, Ludwig Maximilian University of Munich, Pettenkoferstr. 9a, 80336 Munich, Germany; 3 German Center for Infection Research (DZIF), Partner Site, Munich, Germany; 4 Laboratory for Functional Genome Analysis, Gene Center, Ludwig Maximilian University of Munich, Munich, Germany; 5 Department of Medicine III, LMU University Hospital, LMU Munich, Munich, Germany; 6 Department of Medicine I, LMU University Hospital, LMU Munich, Munich, Germany; 7 Helmholtz Munich, Munich, Germany; 8 German Cancer Consortium (DKTK), Partner Site Munich, Munich, Germany

**Keywords:** COVID-19, Solid organ transplantation, Immune responses, Immunosuppression, Lung transplant recipients

## Abstract

**Purpose:**

Lung transplant recipients are at increased risk of severe disease following infection with severe acute respiratory syndrome coronavirus type 2 (SARS-CoV-2) due to high-dose immunosuppressive drugs and the lung is the main organ affected by Coronavirus disease 2019 (COVID-19). Several studies have confirmed increased SARS-CoV-2-related mortality and morbidity in patients living with lung allografts; however, detailed immunological studies of patients with SARS-CoV-2 infection in the early phase following transplantation remain scarce.

**Methods:**

We investigated patients who were infected with SARS-CoV-2 in the early phase (18–103 days) after receiving double-lung allografts (*n* = 4, LuTx) in comparison to immunocompetent patients who had not received solid organ transplants (*n* = 88, noTx). We analyzed SARS-CoV-2-specific antibody responses against the SARS-CoV-2 spike and nucleocapsid proteins using enzyme-linked immunosorbent assays (ELISA), chemiluminescence immunoassays (CLIA), and immunoblot assays. T cell responses were investigated using Elispot assays.

**Results:**

One LuTx patient suffered from persistent infection with fatal outcome 122 days post-infection despite multiple interventions including remdesivir, convalescent plasma, and the monoclonal antibody bamlanivimab. Two patients experienced clinically mild disease with prolonged viral shedding (47 and 79 days), and one patient remained asymptomatic. Antibody and T cell responses were significantly reduced or undetectable in all LuTx patients compared to noTx patients.

**Conclusion:**

Patients in the early phase following lung allograft transplantation are vulnerable to infection with SARS-CoV-2 due to impaired immune responses. This patient population should be vaccinated before LuTx, protected from infection post–LuTx, and in case of infection treated generously with currently available interventions.

**Supplementary Information:**

The online version contains supplementary material available at 10.1007/s15010-023-02116-6.

## Introduction

Transplant recipients (TRs) have a higher risk of contracting infectious diseases due to the use of immunosuppressive agents during the early phase after transplant (< 12 months) [1, 2]. In fact, infections are the leading cause for increased mortality in the first year post-transplantation [3–6]. Bacterial and viral pneumonia are common in Lung Transplant Recipients (LTRs) since the allograft is directly exposed to the pathogen. It is therefore important to find the suitable balance between immunosuppressive rejection prophylaxis and preservation of protective immune responses. While total cell counts for CD4 and CD8 T cells in LTRs seem to remain stable, their function measured as cytokine secretion is significantly impaired [7, 8]. Conversely, humoral immunity is impaired with lower IgG titers especially in the early phase post-solid organ transplantation (SOT) [9, 10]. This is confirmed in recent studies of immune responses to SARS-CoV-2 vaccination, even after a longer time post-transplantation and especially for infection with later variants of concern (VOCs) like omicron [11–13].

LTRs therefore have a high susceptibility for airborne viral infections of the respiratory system such as SARS-CoV-2 [14]. Several studies have confirmed increased morbidity and mortality of LTRs due to COVID-19 in comparison to the general population [13, 15–18]. Risk factors for severe disease include advanced age, male sex, impaired kidney function, and time point of infection following SOT with patients infected at a later time following SOT showing fewer clinical effects than patients with early-onset infections.

Since the outbreak of the COVID-19 pandemic, only few cases of early-onset infection with SARS-CoV-2 have been described in LTRs as clinical case reports without further investigation of their antiviral immune responses. The aim of this study is therefore to investigate the clinical course and SARS-CoV-2-specific immune responses of four LTRs with early-onset infection (LuTx) in comparison to COVID-19 patients who have not received an allograft (noTx).

## Methods

### Study design and subjects

This study is a retrospective monocenter case–control analysis of early-phase lung transplant recipients (LuTx) and non-transplant patients with SARS-CoV-2 infection (noTx). From the day of the WHO declaration classifying COVID-19 as a global pandemic on March 16, 2020 until March 3, 2021, 87 patients underwent a lung transplant in the Munich Lung Transplant Group (MLTG). Infection with SARS-CoV-2 was detected in six of these patients in the early phase after transplant (< 6 months, 22–103 days). Four of these patients, subsequently referred to as LuTx A-D, gave written informed consent for participation in this study.

Patients were recruited in the COVID-19 Registry of the LMU University Hospital Munich (CORKUM, WHO trial ID DRKS00021225). Patient data were anonymized for analysis, and this study was approved by the local ethics committee (Institutional Review Board) (No: 20-245).

For comparison of humoral and cellular immune responses, we selected 88 non-transplant patients (noTx) without documented medical conditions associated with significant immunodeficiency. These patients were part of the CORKUM study and cryopreserved samples were used retrospectively for analysis.

### SARS-CoV-2 RNA detection and quantification

Nasopharyngeal swab samples (ESwab, Copan Diagnostics, Murrieta, USA) were collected twice weekly for patients on the normal ward and transported to the accredited routine diagnostics laboratory of the Max von Pettenkofer Institute. PCR tests were performed using the Roche Cobas SARS-CoV-2 assays on the Cobas 6800 system. Viral load results were calculated as copies per ml of transport medium for the E-gene reaction as described previously [19].

### SARS-CoV-2 whole genome sequencing

Amplicon pools covering the SARS-CoV-2 genome were prepared according to the ARTIC network nCoV-2019 sequencing protocol v2 and analyzed utilizing the ARTIC bioinformatics protocol as described previously [20]. The consensus sequences and associated sample metadata were uploaded to the GISAID repository.

### Antibody detection assays

The commercial recomLine SARS-CoV-2 IgG line immunoassay (Mikrogen, Neuried, Germany) was used to analyze IgG antibodies against the SARS-CoV-2 spike receptor binding domain (RBD) and nucleocapsid (N). Quantitative results were obtained by analyzing test strips with the recomScan software. According to the manufacturer’s guidelines, the “fold cut-off” value was determined by subtracting the signal of interest with that of the internal cut-off band. IgG antibodies against the spike S1 subunit were quantified using the commercial ELISA by Euroimmun (Lübeck, Germany). Nucleocapsid-specific IgG was analyzed using the Abbott SARS-CoV-2 IgG assay (Abbott Diagnostics, Abbott Park, Illinois, United States). All these tests were performed following the manufacturer’s instructions on cryopreserved serum samples collected at the indicated time points.

### ELISPOT analysis

IFN-gamma ELISPOT assays were performed with cryopreserved patients’ PBMCs according to the manufacturer’s recommendations (Mabtech, Nacka, Sweden; Bio-Rad, Puchheim, Germany). Frozen PBMCs were thawed and incubated at 2.5 × 10^5^ cells/well with SARS-CoV-2 peptide pools (Wuhan-Hu-1 PepMix, JPT, Berlin, Germany), consisting of 15mer peptides with 11 amino acid overlap, at a final concentration of 0.5 μg/ml per peptide for 14–18 h. A stimulation cocktail of phorbol 12-myristate 13-acetate (PMA) and ionomycin (0.5X, Thermo Fisher Scientific, Waltham, United States) was used as positive control. Conditions without peptide stimulation serve as negative control and were subtracted from the sample values. Due to limited sample availability, each condition was tested in a single reaction.

### Statistical analysis

Statistical analyses were performed using GraphPad Prism version 9.5 software. Groupwise comparisons were done using Mann–Whitney test. For summary visualization of serological results of noTx patients, locally estimated scatterplot smoothing (LOESS) was performed using the ggplot package in RStudio version 1.2.5033 with the geom_smooth function.

## Results

### Clinical course of SARS-CoV-2 infection in recent lung transplant recipients

We investigated four lung allograft recipients who experienced infection with SARS-CoV-2 in the early phase post-transplantation (18–103 days after transplantation). Clinical characteristics of these patients are summarized in Table 1 and described more detailed in supplementary information as well as in a previously published paper by Zimmermann et al. who investigated the clinical course of patient A and patient B of this study [18].

**Table 1:** Patient characteristics and follow-up

	Patient A	Patient B	Patient C	Patient D
General characteristics				
Age (y)	66	59	58	68
Gender	f	m	m	m
BMI (kg/m^2^)	28	27,7	28	26
Smoking history (PY)	0	100	5	0
Survival	Yes	No	Yes	Yes
Survival (months post-LuTx)	33	4	34	36
Survival SARS-CoV-2 free (months)	32	NA	28	31
Vaccination against SARS-CoV-2	4x	NA	3x	4x
Pre-exposition prophylaxis	Evusheld 1x	NA	Evusheld 1x (booster scheduled)	Evusheld 1x
Underlying disease	EAA (Hypersensitivity pneumonitis)	UIP	ILD	IPF
Comorbidities	HP gastritis coronary atherosclerosis	Alcohol abuse Nicotine abuse	Arterial hypertension coronary atherosclerosis	Arterial hypertension atrial fibrillation
Type LuTx	double	double	double	double
Immunosuppression	Tacrolimus, mycophenolate mofetil, prednisolone	Tacrolimus, mycophenolate mofetil, prednisolone	Tacrolimus, mycophenolate mofetil, prednisolone	Tacrolimus, switch to cyclosporine A, mycophenolate mofetil, prednisolone
Graft function				
Humoral rejection (HLA-DSA)	NA	NA	NA	15.09.2020 A24, -B8, -Cw9 MFI 3800
Cellular rejection	NA	28.10.2021 A1, B0	NA	NA
Treatment of rejection	NA	high-dose corticosteroids	NA	IVIG ECP (ongoing)
Latest FEV1 (L/% of best)	1.78 (99)	NA	2.07 (100)	1.69 (84%)
Time after Tx to SARS-CoV-2-positive PCR (d)	18	22	94	103
COVID severity and mortality risk factors				
Renal function (minimal GFR during SARS-CoV-2)	20	12	39	14
(maximum serum creatinine mg/dl)	2.4	4.9	1.9	4.1
D-dimer (µg/ml)	0.6	2.9	1	0.5
Obesity (BMI > 25)	Yes	Yes	Yes	Yes
Diabetes	No	No	No	No
Hypertension	No	No	Yes	Yes

*BMI* body mass index, *y* years, *py* pack years, *UIP* unspecified interstitial pneumonia, *EAA* extrinsic allergic alveolitis/hypersensitivity pneumonitis, *ILD* interstitial lung disease, *IPF* idiopathic pulmonary fibrosis, *LuTx* lung transplantation, *FEV1* Forced expiratory volume in 1 s

**Fig. 1:**
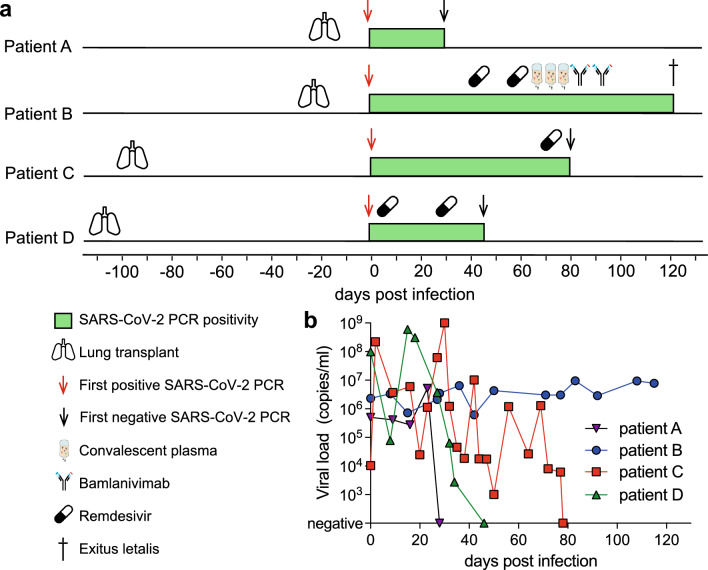
**a** Clinical course of SARS-CoV-2 infection of recent lung transplant recipients. The timeline indicates the order of events in relation to the date of the first positive SARS-CoV-2 PCR result (day 0, red arrow). The symbols indicate the time points of administration of remdesivir (pills), convalescent plasma (infusion bag), and bamlanivimab (monoclonal antibody), respectively. The first negative SARS-CoV-2 PCR result (black arrow) is indicated. Patient B passed away after 121 days of infection (cross). **b** SARS-CoV-2 viral load trajectories of recent lung transplant recipients. Viral load is indicated as copy numbers of the SARS-CoV-2 ORF1ab gene per ml of transport medium of nasopharyngeal swab samples. Values are plotted in relation to the first positive PCR result (days of infection) for each of the four transplant recipients

Remarkably, the spectrum of SARS-CoV-2-induced disease ranged from asymptomatic infection with rapid clearance (patient A) to persistent infection with ultimately fatal outcome (patient B). The clinical course and viral load results from respiratory samples in relation to the time point of transplantation are illustrated in Fig. 1a, b.

Patient A cleared the infection with negative PCR results from 31 DPI and never developed COVID-19-related symptoms.

Despite multiple therapeutic approaches including remdesivir, transfusion of convalescent plasma [three consecutive infusion regimens with 2 × 200 ml administration of COVID convalescent plasma (CCP)], and application of the monoclonal antibody bamlanivimab, patient B remained PCR positive. He developed pulmonary and gastrointestinal symptoms including diarrhea. He was transferred to the intensive care unit 95 days after infection. Forth following, he developed hepatopathy with subsequent liver failure alongside intermittent renal failure with renal replacement therapy for 7 days, while experiencing worsening lung affection showing consolidations matching viral pneumonia with bacterial superinfection. Respiratory failure required intubation and mechanical ventilation from day 102 to death. (Fig. 1b). Patient C presented with mild symptoms (elevated temperature but no fever, mild dyspnea with oxygen therapy, and only marginal infiltrates in chest CT). Due to extended therapy for cytomegalovirus (CMV) reactivation (see supplementary material), he developed leukopenia. As no viral clearance could be achieved, he received remdesivir therapy on DPI 72–78 and tested negative after 79 DPI. Patient D developed progredient bilateral pulmonary ground-glass lesions as well as mild disease symptoms. He also developed acute renal failure (max. creatinine levels 4.1 mg/dl, minimum GFR 14) without need for renal replacement therapy. As he experienced increasing symptoms (dyspnea, need for oxygen therapy, fatigue) alongside pulmonary affection in CT scans, he received dexamethasone alongside two therapy regimens of remdesivir (DPIs 2–6 and 28–32). He cleared the infection on 47 DPI.

All three survivors are being followed up closely by our LuTx program. They do not show signs of long COVID. All received at least three vaccinations against SARS-CoV-2 alongside tixagevimab/cilgavimab (Evusheld) for pre-exposure prophylaxis. None experienced another infection with SARS-CoV-2. The latest anti-SARS-CoV-2 S antibodies were 8.7 for patient A, 2.9 for patient C, and 8.5 for patient D (U/ml; IgG; Euroimmun; cut-off < 0.8).

Patient D developed CLAD type BOS [humorally triggered by donor-specific antibodies (DSAs)]. Therapy with intravenous immunoglobulins (IVIG) was not successful in clearing the DSA, so that he is currently under extracorporeal photopheresis therapy (ECP, currently 22 treatment regimens). Patients A and C did not develop CLAD.

Survival and graft function along with current treatment and further clinical data are also summarized in Table 1. No patient in our cohort underwent induction therapy prior to LuTx.

As mentioned above, in a previous detailed report by Zimmermann et al. [18] focusing on clinical course of disease, more in-depth information on the same patients has been described. This includes further data on comorbidities, transplant specifics like HLA typing, and overall clinical course of SARS-CoV-2 and post-transplantation period, but limited data on immune responses reporting SARS-CoV-2-specific antibodies without distinguishing between nucleocapsid- and spike-specific responses and quantity.

In this study we aimed to decipher SARS-CoV-2-specific immune responses in these four LuTx cases with differential disease outcome and in comparison to noTx patients.

### Limited antibody responses following SARS-CoV-2 infection in recent lung transplant recipients

**Fig. 2:**
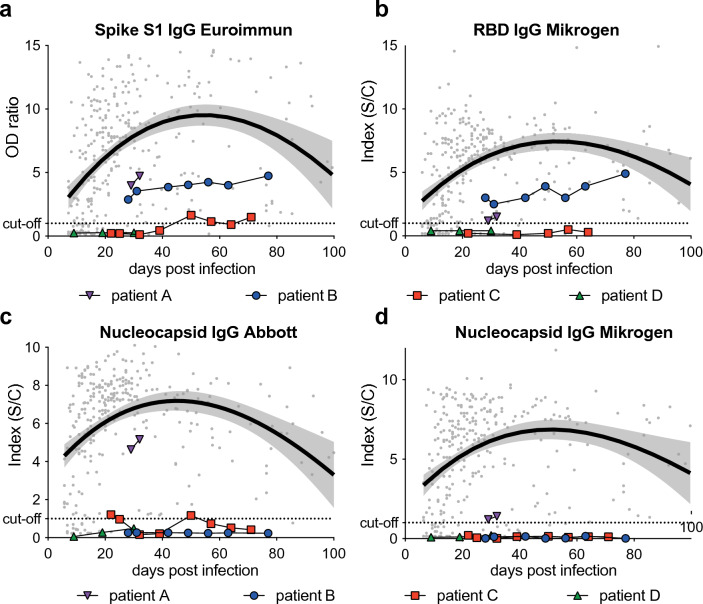
Limited SARS-CoV-2-specific antibody responses in lung transplant recipients in comparison to non-immunocompromised patients. Serological results are shown for four commercially available assays for the detection of IgG antibodies against the SARS-CoV-2 spike S1 subunit (**a**), the receptor binding domain (RBD) (**b**) and nucleocapsid (**c**, **d**). Longitudinal results for the four lung transplant recipients (indicated in color) are shown in relation to the first positive SARS-CoV-2 PCR result. For comparison, longitudinal serological results of patients who have not received a solid organ transplant (noTx *n* = 88) are plotted as gray dots with locally estimated scatterplot smoothing (LOESS) shown as black curve with 95% confidence interval. Results are indicated as optical density for the chemiluminescence assay (**a**), or signal to cut-off ratio for immunoblot assays (**b–d**). The dotted horizontal line represents the cut-off considered for test result positivity as provided by the manufacturer

To investigate the effect of iatrogenic immunosuppression in the early post-transplantation phase in lung allograft recipients on antibody responses upon SARS-CoV-2 infection, we measured IgG antibodies against spike and nucleocapsid antigens over time (Fig. 2). In comparison to the lung transplant recipients (LuTx), we tested 309 longitudinal samples from 88 patients without documented causes for immunosuppression from the local COVID-19 cohort, the COVID-19 registry of the LMU clinic (CORKUM) (noTx). Clinical characteristics of these control patients are summarized in the Supplementary Information (SI).

Compared to these control patients, LuTx showed generally lower levels of SARS-CoV-2-specific antibodies. Patient B had detectable IgG targeting the spike S1 subunit and receptor binding domain (Fig. 2a, b).

Of note, longitudinal sequence analysis of viral isolates from this patient revealed that the well-characterized antibody escape mutation at spike residue position 484 in the receptor binding domain (RBD) (E484K) had emerged as de novo mutation at 42 DPI. This may have resulted in reduced neutralizing efficacy of these autologous antibodies and the administered convalescent plasma and therapeutic monoclonal antibody, bamlanivimab, possibly contributing to viral persistence. Patient A who cleared the infection relatively quickly had detectable antibodies to the spike and nucleocapsid antigens, whereas the other patients only showed negative or borderline reactivity.

### Dampened T cell responses against SARS-CoV-2 in the early post-transplantation phase of lung allograft recipients

**Fig. 3:**
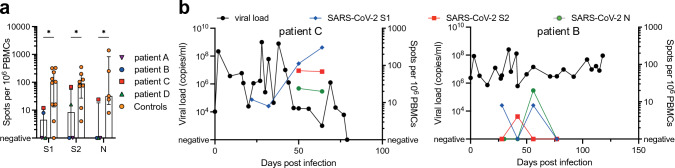
Impaired T cell responses against SARS-CoV-2 in lung transplant recipients compared to non-immunocompromised controls.** a** PBMC samples of the four lung transplant recipients were stimulated with peptide pools covering the S1 and S2 subunits of the SARS-CoV-2 spike protein and the nucleocapsid protein (N). Results are shown as spots per million PBMCs for the time point closest to 30 days post-infection if multiple time points were available (patient A: 16, patient B: 28, patient C: 32 for S1 and 50 for S2 and N, patient D: 29 days post-infection). For comparison, non-immunocompromised donors were tested matched to 30 days post-infection for S1 (*n* = 10), S2 (*n* = 8) and N (*n* = 5) Elispot responses (blue). Bar graphs represent median values with interquartile range. Groupwise comparisons were performed using the Mann–Whitney test with * indicating *p*-values < 0.05.** b** Longitudinal T cell responses against S1 (blue), S2 (red), and N (green) peptide pools are shown for patient C and patient B in relation to viral load (black)

The immunosuppressive regimen used in the early phase post-transplantation consists of tacrolimus, mycophenolate mofetil, and prednisolone to prevent allograft rejection. To investigate the effect of this potent combination on T cell responses against SARS-CoV-2, we analyzed Elispot responses of PBMCs of the infected transplant recipients against peptide pools covering the S1 and S2 subunits of the SARS-CoV-2 spike protein and the nucleocapsid protein (N) of the Wuhan-Hu-1 reference strain. In comparison we tested samples of ten SARS-CoV-2-infected immunocompetent donors. Due to sample availability, we were only able to test the S1 antigen for all ten control patients and the S2 and N antigen for a subset of these (*n* = 8 and *n* = 5, respectively). Since the magnitude of T cell responses expands and contracts over time after antigen contact, we selected samples to match time after infection around 30 DPI (20–45 DPI for control patients). As expected, the magnitude of the T cell response in transplant recipients (LuTx) was lower compared to the control patients for all three antigens (*p* = 0.031, *p* = 0.046, and *p* = 0.039 for S1, S2, and N, respectively) (Fig. 3a). For patients B and C we had longitudinal samples available for further testing. Interestingly, for patient C we detected robust T cell responses against the three antigens shortly before viral clearance, whereas for patient B we only detected transient responses at lower magnitude consistent with persistent viral replication (Fig. 3b).

## Discussion

The findings of this study and in-depth analysis of immune responses to SARS-CoV-2 in severely immunocompromised patients early after lung transplantation emphasize the special precautions that still have to be kept in place for this vulnerable population, even though the pandemic has been declared over.

Our findings are consistent with previously published data by Hodge et al. [8], highlighting impaired immune responses to SARS-CoV-2 infection of LTRs treated with high-dose immunosuppressants through decreased T cell activity. Further groups have shown that LTR under high levels of immunosuppressive drugs can present severe COVID-19 course of disease and experience longer periods of viral shedding compared to those with lower levels of immunosuppression [21, 22]. We were able to confirm these findings by comparing our at risk group with a large cohort of immunocompetent subjects demonstrating impaired cellular and humoral immune responses.

Our patient cohort presented with several risk factor combinations affecting severity of disease as well as mortality. Consistent with literature [23–25] male gender, multiple comorbidities like diabetes or hypertension, smoking history, obesity, and acute kidney injury are in line with the differential disease severity and mortality observed in our cases, although the sample size of our study was insufficient to formally assess risk factors.

Our study has several important limitations. This study was performed during the first wave and second wave of the pandemic in Germany before SARS-CoV-2 vaccines became available, therefore precluding extrapolations of our findings to the current situation with the vast majority of the population having built up immunity to SARS-CoV-2 by vaccination or infection or most often a combination of both. Also, our study is limited to only four at risk patients due to the fact that the occurrence of SARS-CoV-2 infection in the early phase following transplantation was fortunately rare.

Only one of the investigated patients, patient B, was unable to clear the infection and passed away. In this particular case limited and transient CD8 T cell responses and potentially the emergence of the antibody immune escape mutation S:E484K may have contributed to persistent infection with an ultimately fatal outcome [26]. Of note, despite the very low to undetectable levels of anti-SARS-CoV-2 antibodies in patients C and D, both patients cleared the infection successfully. SARS-CoV-2-specific T cell responses were detected in both individuals in line with other studies that demonstrate the importance of T cell immunity for viral clearance in animal models and in patients with deficient antibody responses [27–29].

Several factors may contribute to the inability of patients with high levels of immunosuppression to clear SARS-CoV-2 infections, including the type and dosage of immunosuppressive drugs, the duration of treatment, and the presence of comorbidities. In the case of patient B, convalescent plasma therapy failed to induce viral clearance. Since convalescent plasma therapy was applied as early as available in our center, little data were available at that time on the amount to be transfused and in what phase of infection. More recent data recommend convalescent plasma for early disease and immunosuppressed patients [30]. Of note, anti-SARS-CoV-2 antibody levels in patient B did not increase significantly after the administration of convalescent plasma potentially due to low levels of specific antibodies in the applied plasma transfusions. Further research suggests higher efficacy in dependence of the SARS-CoV-2-specific antibody content of the administered convalescent plasma therapy [31]. Therapeutic monoclonal antibodies targeting SARS-CoV-2 are another important option in the pre-exposure prophylaxis and treatment of immunocompromised patients, but it has to be considered that their neutralizing capacity may be limited against currently circulating variants as recently observed for various Omicron sublineages [32].

Overall, it is crucial to carefully monitor and case-dependent eventually isolate lung transplant recipients in the early phase after transplantation. In addition, in the case of SARS-CoV-2 infection it is vital to adjust their immunosuppressive treatment when necessary to prevent the development of severe COVID-19 disease and reduce the risk of viral persistence, as well as the emergence of new viral variants. It is crucial to strike a balance between preventing organ rejection and maintaining a robust immune response against infectious agents, particularly in the context of COVID-19.

### Supplementary Information

Below is the link to the electronic supplementary material.

Supplementary file1 (DOCX 16 KB)

## Data Availability

Data sets and material can be made available on request if applicable.
