# Supplementary material for: Impaired immune responses and prolonged viral replication in lung allograft recipients infected with SARS-CoV-2 in the early phase after transplantation
Source: Infection. 2023 Nov 3;52(3):847–55. doi: 10.1007/s15010-023-02116-6 (PMC11143031; doi:10.1007/s15010-023-02116-6)
Supplement: Supplementary file 1 — Supplementary file1 (DOCX 16 KB) [file 15010_2023_2116_MOESM1_ESM.docx]

SUPPLEMENTARY Information

1. Detailed description of clinical courses of LuTx patients

Patient A is a 66-year old female patient with EAA. She had no smoking history but showed low-grade coronary atherosclerosis and an Helicobacter pylori (H.pylori) positive gastritis. She underwent uneventful DLuTx without the need for ECMO 18 days before SARS-CoV-2 infection was detected. The clinical course post-LuTx was uneventful and she was transferred to the normal ward after six days of intensive care. She recovered quickly and was already fully mobile and on a normal diet at the time of the first positive swab for SARS-CoV-2. Subsequent rRT-PCRs performed on DPIs 9, 16 and 23 resulted positive. She never presented symptoms of Covid-19 and on DPI 31 she tested negative.

Patient B was a 59-year old male patient with Usual Interstitial Pneumonia (UIP). He had a smoking history of 100 pack-years (PY) and a history of alcohol abuse, but no other comorbidities. He received a double-lung transplantation (DLuTx) on central veno-arterial Extracorporeal Membrane Oxygenation (ECMO) 22 days before the first positive SARS-CoV-2 test result. After only three days of intensive care after transplantation, he was transferred to the normal ward. Since standard transbronchial biopsy at reduced corticosteroid doses revealed mild cellular rejection (A1B0, according to ISHLT (44)), he underwent short-time high-dose intravenous corticosteroid treatment shortly before the first SARS-CoV-2 positive swab was taken. Remdesivir was administered during a period of four days on two occasions: DPIs 44 – 48 and 64 – 68. Three doses of convalescent plasma were administered on DPIs 73, 75 and 78. Finally, Bamlanivimab was administered twice on DPIs 86 and 87. Over the course of disease on the normal ward, he developed pulmonary and gastrointestinal symptoms including diarrhoea. SARS-CoV-2 was also detected in excretions. Forth following, he developed hepatopathy with subsequent liver failure alongside intermittent renal failure with renal replacement therapy for 7 days, while experiencing worsening lung affection (consolidations matching viral pneumonia to bacterial superinfection). He was transferred to the intensive care unit on DPI 95. Respiratory failure required intubation and mechanical ventilation from day 102 to death on DPI 122.

Patient C is a 58-year old male patient with a smoking history of 5 PY. He presented for LuTx with arterial hypertension and coronary atherosclerosis. Due to increasing hypoxemic respiratory insufficiency, he was put on veno-venous extracorporeal membrane oxygenation (VV-ECMO) as a bridge-to-transplant 14 days prior to LuTx, until three days post-LuTx. After surgical revision for hemothorax and 38 days of intensive care for continuous respiratory insufficiency he was successfully transferred to the normal ward. The course on the normal ward was affected by post-ICU critical illness polyneuropathy and consecutive difficulties in mobilization and motivation.

After testing positive for SARS-CoV-2 he developed only mild symptoms (elevated temperature but no fever, mild dyspnea with oxygen therapy, only marginal infiltrates in chest CT). As an additional problem, laboratory checkups revealed cytomegalovirus reactivation that led to switch of therapy from Aciclovir to Valganciclovir and eventually iv Ganciclovir. Unfortunately, this led to leukopenia, so that therapy was discontinued. Given continuously PCR-positive swabs, he received remdesivir treatment once for a period of 5 days (DPIs 72 – 78).

Further postoperative complications were a perforation of a gastric ulcer and consecutive emergency surgery with suture of the perforation site. Eventually, he tested negative for Sars-CoV 2 after 79 days.

Patient D is a 68-year old male patient with interstitial pulmonary fibrosis and no smoking history. Pretransplantation BMI was normal and the only comorbidities were medically well-controlled arterial hypertension and intermittent atrial fibrillation. He received DLuTx on veno-arterial ECMO for mPAP of >35mmHg. The Postoperative course in the ICU was affected by neurological impairment and delirious state, prolonging ICU to around 6 weeks. As a means to lessen drug-induced neurological impairment, he was intermittently put on cyclosporine A and tacrolimus was discontinued. This was changed back after ruling-out organic brain causality for neurological impairment. An episode of mild rejection (continuous oxygenation impairment) with detection of low-level donor specific antibodies (Anti-HLA-A24, -B8, -Cw9), was treated with four days of iv immunoglobuline (Pentaglobin). For prolonged weaning the patient underwent tracheostomy nine days post-LuTx. Tracheostomy remained for 29 days. After 41 days on the ICU, he was eventually transferred to the normal ward. Apart from re-detection of above-mentioned HLA-Antibodies that were again treated with three days of iv immunoglobulin, his stay on the normal ward was uneventful and he was discharged nine weeks post-LuTx. He was diagnosed as Sars-CoV2 positive in an external University hospital on 15 November 2020 and readmitted to our institution's COVID isolation ward two days later. He presented with renal failure most likely caused by tacrolimus overdose. CT showed progredient bipulmonal ground glass lesions typical for Covid-19 and he developed mild symptoms (dyspnea, need for oxygen therapy, fatigue) and received remdesivir twice for a period of four days each (DPIs 2 – 6 and 28 – 32). Over the course of hospitalisation the patient also developed atypical hemolytic uremic syndrome and immunosuppressive therapy was changed to cyclosporine. Sars-CoV2 PCR results were negative from 47 DPI and the patient was eventually discharged in good clinical state to home care.

1. Clinical characteristics of control patients

Control patients were recruited during the first wave of the pandemic between March and June 2020 in the CORKUM study. A total of 309 longitudinal samples from 88 individual patients were investigated for SARS-CoV-2 specific IgG responses. These control patients were selected to have not received solid organ transplants or other underlying medical conditions associated with significant immunosuppression from their medical records. The median age of this control group was 62 years (51-72 years interquartile range); 66 were male and 22 female; COVID-19 disease severity as assessed using the WHO severity score was 1 for n=7 (ambulatory, no limitation of activities), 2 for n=2 (ambulatory, limitation of activity), 3 for n=33 (hospitalized mild disease, no oxygen), 4 for n=14 (hospitalized mild disease, oxygen by mask or nasal prongs), 5 for n=2 (hospitalized severe disease, non-invasive ventilation or high-flow oxygen), 6 for n=17 (severe disease, intubation and mechanical ventilation), 7 for n=2 (severe disease, ventilation and additional organ support) and 8 for n=11 (lethal outcome).
